# Serum Clusterin as a Tumor Marker and Prognostic Factor for Patients with Esophageal Cancer

**DOI:** 10.1155/2014/168960

**Published:** 2014-12-10

**Authors:** Wei Guo, Xiao Ma, Christine Xue, Jianfeng Luo, Xiaoli Zhu, Jiaqing Xiang, Bo Lu, Hecheng Li

**Affiliations:** ^1^Department of Thoracic Surgery, Fudan University Shanghai Cancer Center (FUSCC), Shanghai 200032, China; ^2^Department of Oncology, Shanghai Medical College, Fudan University, Shanghai 200032, China; ^3^Department of Radiation Oncology, Kimmel Cancer Center and Jefferson Medical College of Thomas Jefferson University, Philadelphia, PA 19107, USA; ^4^Department of Biostatistics, Public Health School, Fudan University, Shanghai 200032, China; ^5^Department of Pathology, Fudan University Shanghai Cancer Center (FUSCC), Shanghai 200032, China; ^6^Department of Thoracic Surgery, Ruijin Hospital, Shanghai Jiaotong University School of Medicine, 197 Ruijin 2nd Road, Shanghai 200025, China

## Abstract

*Background*. Recent studies have revealed that clusterin is implicated in many physiological and pathological processes, including tumorigenesis. However, the relationship between serum clusterin expression and esophageal squamous cell carcinoma (ESCC) is unclear.* Methods*. The serum clusterin concentrations of 87 ESCC patients and 136 healthy individuals were examined. An independent-samples Mann-Whitney *U* test was used to compare serum clusterin concentrations of ESCC patients to those of healthy controls. Univariate analysis was conducted using the log-rank test and multivariate analyses were performed using the Cox proportional hazards model.* Results*. In healthy controls, the mean clusterin concentration was 288.8 ± 75.1 *μ*g/mL, while in the ESCC patients, the mean clusterin concentration was higher at 412.3 ± 159.4 *μ*g/mL (*P* < 0.0001). The 1-, 2-, and 4-year survival rates for the 87 ESCC patients were 89.70%, 80.00%, and 54.50%. Serum clusterin had an optimal diagnostic cut-off point (serum clusterin concentration = 335.5 *μ*g/mL) for esophageal squamous cell carcinoma with sensitivity of 71.26% and specificity of 77.94%. And higher serum clusterin concentration (>500 *μ*g/mL) indicated better prognosis (*P* = 0.030).* Conclusions*. Clusterin may play a key role during tumorigenesis and tumor progression of ESCC and it could be applied in clinical work as a tumor marker and prognostic factor.

## 1. Background

Esophageal squamous cell carcinoma (ESCC) is one of the most lethal cancers worldwide, especially in developing countries like China [1, 2]. Despite great advances in surgery, radiotherapy, and chemotherapy, the overall 5-year survival rate for ESCC patients is as low as 36% [3]. Advanced ESCC patients have a poor prognosis and low quality of life. For early detection and diagnosis of ESCC, it is critical to discover new tumor markers from body fluids to delineate tumorigenesis and discriminate between cancer stages.

Clusterin, which was originally identified as serum apolipoprotein J [4], has more recently been found to be expressed widely in human tissues and body fluids and to play an important role in tissue remodeling, reproduction, lipid transport, complement regulation, and apoptosis [5]. There are two subtypes of clusterin in human fluids and tissues: secreted clusterin (sCLU) and nuclear clusterin (nCLU) [6]. The aim of our study was to investigate the serum sCLU expression in ESCC patients and to examine the prognostic value of clusterin.

To achieve this goal, we analyzed the survival data of 87 ESCC patients who underwent radical resection of esophageal carcinoma and determined the preoperative serum sCLU concentration. As a control, serum sCLU concentrations in 136 healthy individuals were also examined.

## 2. Materials and Methods

### 2.1. Patient Selection and Serum Specimens

Ninety-four consecutive patients underwent radical resection of esophageal carcinomas from April 16 to September 2, 2009, at the Fudan University Shanghai Cancer Center. All 94 patients were diagnosed with esophageal cancer by endoscopy or pathology report. Each patient received an esophageal contrast barium study, computed tomography of the chest, and ultrasonography of the abdomen prior to surgery. None of them received preoperative neoadjuvant chemoradiotherapy. Excluding one esophageal adenocarcinoma patient, one neuroendocrine carcinoma patient, two compound carcinoma patients, one patient with uncertain pathology, and two patients without a clusterin concentration report, the remaining 87 ESCC patients were included in this study after receiving their paraffin pathology reports. The Institutional Review Board of the Fudan University Shanghai Cancer Center approved the use of the esophageal carcinoma database for the present study.

Presurgical serum specimens were obtained with written informed consent from all 87 ESCC patients and 136 healthy controls. All the 87 patients were newly pathologically diagnosed as ESCC and previously untreated. Serum was obtained at the time of diagnosis and kept frozen at −80°C until being used. The concentration of clusterin was analyzed by an independent contracted laboratory using an enzyme-linked immune sorbent assay (ELISA) method. All serum specimens were thawed only once before analysis and the diluted serum specimens were analyzed only once.

### 2.2. Follow-Up

After surgery, 80 patients were followed up in the clinic using physical examination, serum tumor marker tests, routine blood tests, esophageal contrast barium study, gastroscopy, neck/chest/abdominal computed tomography, and ultrasonography of the neck and abdomen. Seven patients were lost to follow-up. Follow-up evaluations were conducted every 3 months for the first year, every 4 months during the second year, and every 6 months afterwards. In the 80 patients with follow-up records, the median follow-up period was 27 months (range, 1–60 months).

### 2.3. Statistical Methods

Overall survival was calculated as the length of time from the date of surgery to the date of death or last follow-up visit. Serum clusterin concentrations in ESCC patients and controls were compared using an independent-samples Mann-Whitney *U* test. Survival rates were estimated using the Kaplan-Meier method. Univariate and multivariate analyses were performed using the Cox proportional hazards model. Statistical analyses were carried out with SPSS version 19 (SPSS, Inc., Chicago, IL, USA), GraphPad Prism version 6.01 (GraphPad Software, Inc., La Jolla, CA, USA), and Confidence Interval (CI) Analysis version 2.2.0 (T. Bryant; University of Southampton, UK). A *P* value of <0.05 was considered significant.

## 3. Results

### 3.1. Patient Characteristics

In total, 87 patients, including 79 males and 8 females, were included in this study. The mean age for all patients at the time of surgery was 59.2 ± 8.3 years (range, 37–76). The tumor was located in the upper section of the esophagus in 5 patients, in the middle section in 58 patients, and in the lower section in 25 patients. With regard to pathological stage, the number of patients with stage I, stage II, and stage III cancers was 13, 35, and 39, respectively. The average lymph node yield was 24.3 ± 13.3 (range, 3–83). All patient characteristics are reported in Table 1.

### 3.2. Serum Clusterin Concentration

**Figure 1:**
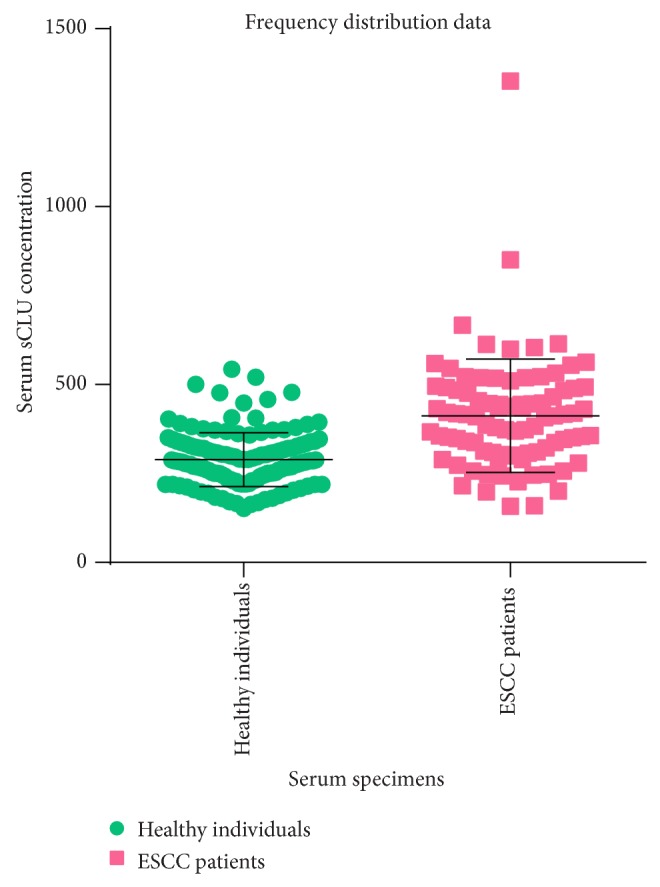
The frequency distribution data of ESCC patients and healthy individuals.

Presurgical serum specimens from 87 ESCC patients and 136 healthy controls were assayed by ELISA. In the ESCC patients, the mean clusterin concentration was 412.3 ± 159.4 *μ*g/mL (range, 158.7–1,352.5), while in healthy controls, the mean clusterin concentration was 288.8 ± 75.1 *μ*g/mL (range, 152.5–543.1). Serum clusterin concentrations in ESCC specimens were significantly higher than those in the control samples (*P* < 0.0001; 95% CI, 84.42 to 141.8). The frequency distribution data of ESCC patients and healthy individuals are shown in Figure 1. Comparisons of ESCC patient and healthy control serum clusterin concentrations are reported in Table 2.

### 3.3. Serum Clusterin as a Tumor Marker for ESCC

**Figure 2:**
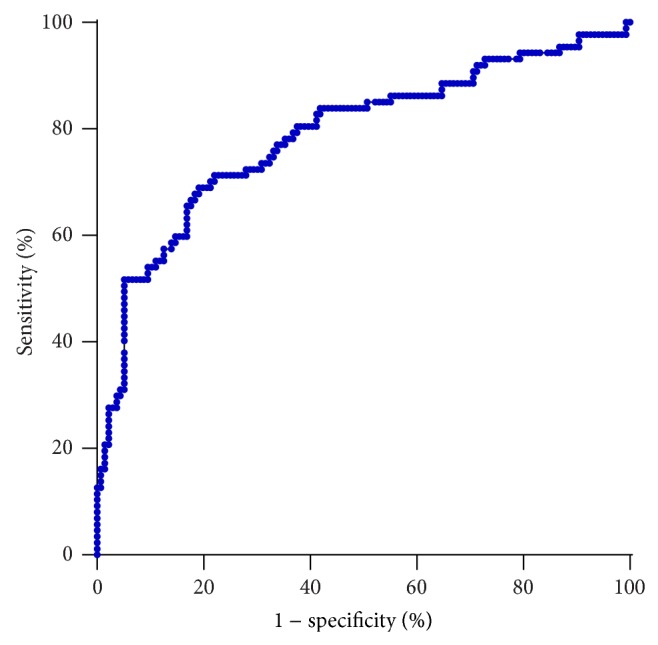
The ROC curve of serum clusterin.

Serum clusterin concentration was significantly higher in ESCC patients. We supposed it as a tumor marker for esophageal squamous cell carcinoma. A receiver operating characteristic curve (ROC curve) was drawn to prove our hypothesis. The ROC curve was shown in Figure 2. As a result, the area under the ROC curve (AUROC) was 0.7873 (*P* < 0.0001; 95% CI, 0.7224 to 0.8521). Optimal sensitivity and specificity of this ROC were calculated using the method reported by Peat and Barton [7]. By our calculation, serum clusterin had an optimal diagnostic cut-off point (serum clusterin concentration = 335.5 *μ*g/mL) for esophageal squamous cell carcinoma with sensitivity of 71.26% (95% CI, 60.57% to 80.46%) and specificity of 77.94% (95% CI, 70.03% to 84.59%).

### 3.4. Prognostic Factors for ESCC Patients

To examine the factors that predict prognosis for ESCC patients, we performed a univariate analysis of the clinicopathological factors (Table 3, (a) and (b)). Among the factors analyzed, lymph node metastasis number and pathological stage were found to be significant prognostic factors (*P* = 0.009 and *P* = 0.001, resp.). Other factors, such as gender, age, tumor location, lymph node yield, and clusterin concentration, were not significantly correlated with prognosis in ESCC patients (Table 3(a)).

To identify independent prognostic factors, four factors were subjected to multivariate analysis: gender, pathological stage, lymph node metastasis number, and clusterin concentration. Multivariate analysis revealed that gender and pathological stage were two independent prognostic factors in ESCC patients. Clusterin concentration was found to be a significant prognostic factor, and patients with a higher clusterin concentration (>500 *μ*g/mL) exhibited a better prognosis than those with lower concentrations (Table 3(b)).

### 3.5. Survival Analysis

**Figure 3:**
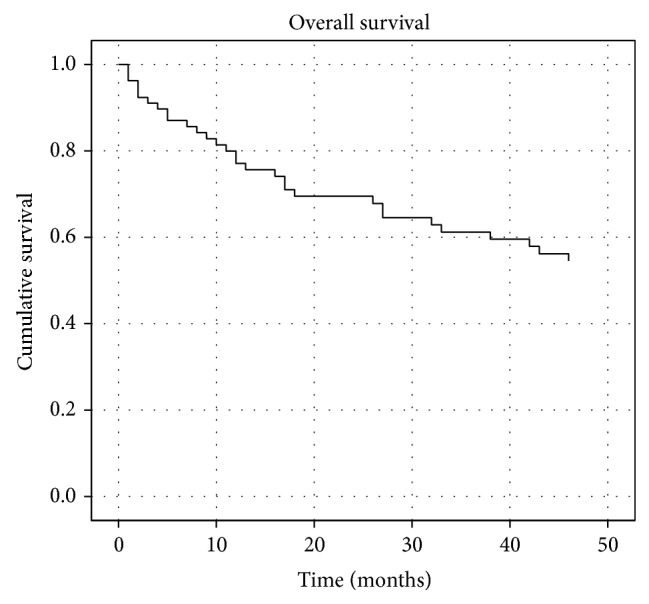
Overall survival curve.

The overall survival curve was analyzed by the Kaplan-Meier method. Our study showed that the 1-, 2-, and 4-year survival rates for the 87 ESCC patients were 89.70%, 80.00%, and 54.50%, respectively (Figure 3).

**Figure 4:**
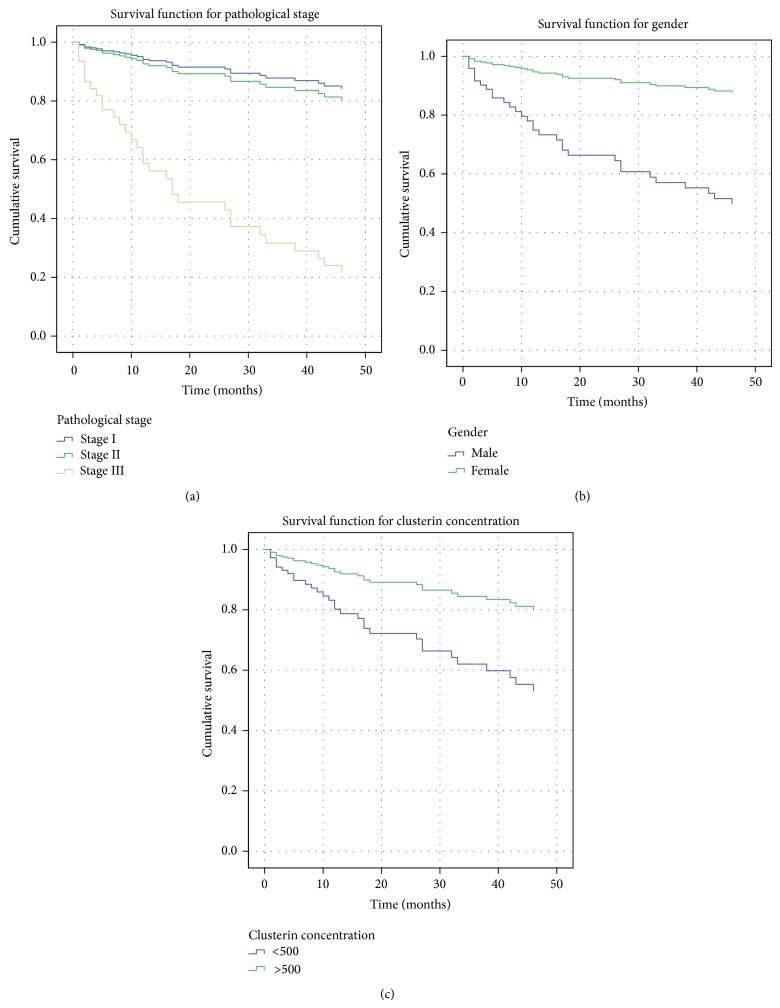
Factors determining overall survival. (a) Survival curves of pathological stage I, stage II, and stage III tumors (*P* = 0.006, *P* < 0.0001). (b) Survival curves for male and female patients (*P* = 0.040). (c) Survival curves for groups of patients with clusterin concentrations of <500 *μ*g/mL and >500 *μ*g/mL (*P* = 0.030).

Pathological stage was an independent prognostic factor for ESCC patients, and significant differences in prognosis between different stages were noted. The *P* values for stage I versus stage III and stage II versus stage III were 0.006 and <0.0001, respectively (Figure 4(a)).

Although gender was not significantly correlated with prognosis of ESCC patients by univariate analysis, we included gender as a factor in multivariate analysis because the *P* value for the comparison of males and females was <0.20. In multivariate analysis, the *P* value of gender (male versus female) was 0.040; therefore, we ultimately regarded gender as an independent prognostic factor for ESCC patients. The survival curves of males and females were shown in Figure 4(b).

Survival curves for groups of patients with clusterin concentrations of <500 *μ*g/mL and >500 *μ*g/mL were shown in Figure 4(c). ESCC patients with higher serum sCLU had a better survival prognosis (*P* = 0.030).

## 4. Discussion

CLU function is considered enigmatic, as it has been associated with various contradictory roles in cellular function, including cell apoptosis, tumorigenesis, and tumor progression [6]. There are two subtypes of clusterin in human fluids and tissues: secreted clusterin (sCLU) and nuclear clusterin (nCLU) [8]. Secreted clusterin functions to eliminate impurities and protect somatic cells from injury, while nCLU induces cell death [9, 10]. Our work focused on the secreted form, as human fluids, such as serum, are an important clinical source for disease markers. Unlike other tumors, such as those of the colon, lung, pancreas, and breast [11–14], the relationship between ESCC and sCLU is currently unclear, and studies in this area are rare [15–17]. Our study, which established an expression model of serum sCLU in ESCC patients, aimed to analyze the expression features of serum sCLU in ESCC patients and the relationship between serum sCLU expression and ESCC patient prognosis.

According to our study, ESCC patients overexpressed serum sCLU compared with healthy controls (*P* < 0.0001). Luo et al. [18] demonstrated that sCLU could protect cells from senescence. In the tumorigenesis stage, the overexpression of clusterin may be a compensatory action to protect somatic cells. The overexpression of sCLU may also be due to tumor cell secretion. Yu and Stamenkovic [19] and Zucker et al. [20] found that matrix metalloproteinase (MMP) plays an important role in neoplasm invasion and metastasis. Interestingly, CLU is a potent promoter of CLU-induced MMP-9 expression, according to Wang et al. [21]. Different pathology stages may also affect the serum sCLU concentration. Our finding directly contradicts that of Zhang et al.'s 2003 study [17], in which clusterin was markedly downregulated in both serum and tissues of ESCC. This apparent discrepancy may also be present in other neoplasms, such as colorectal cancer [22–24]. These diverse findings may stem from differences between clusterin protein subtypes that were not differentiated as a result of the opposing functions of sCLU and nCLU [9, 10, 25]. More specific and accurate detection methods are necessary in order to differentiate sCLU from nCLU and allow comparison of study results. Previous studies showed that smoking, cardiovascular and cerebrovascular diseases, alcohol consumption, and diabetes could enhance the expression of sCLU [26–28]. To reveal the true expression difference caused by ESCC, we also excluded patients with these confounding factors from the ESCC group to compare the sCLU expression between ESCC group and the healthy group; ESCC group still showed significantly higher sCLU expression (Table 2). This finding implied that it is the tumor itself that led to the overexpression of sCLU, rather than smoking, cardiovascular and cerebrovascular diseases, alcohol consumption, or diabetes. In our study, serum sCLU was significantly overexpressed in ESCC patients and at the cut-off point of 335.5 *μ*g/mL, serum sCLU has sensitivity of 71.26% and specificity of 77.94% for the diagnosis of esophageal squamous cell carcinoma. These results indicate that sCLU was a biomarker for ESCC patients. There are currently no specific tumor markers for esophageal squamous cell carcinoma, but if additional studies confirm our result, we can define sCLU as a tumor marker for ESCC.

In our study, clusterin showed no significant association with ESCC prognosis in univariate analysis (*P* = 0.392), but in multivariate and survival curve analysis, sCLU overexpression (>500 *μ*g/mL) was related to better prognosis (*P* = 0.030). ESCC patients have higher sCLU concentration but ESCC patients with higher sCLU concentration have better prognosis; this is an interesting discovery. We suppose that sCLU was overexpressed in ESCC patients according to tumorigenesis but sCLU could also protect normal cells from senescence [18]. In Park et al.'s elaborate review, sCLU can promote survival because of its cardioprotective, antifibrosis, and antidiabetes function [27]. Thus, in our study, ESCC patients with higher sCLU concentration tend to have a better prognosis. Admittedly, more researches are needed to expound the accurate function of clusterin and confirm the diagnostic and prognostic role of sCLU in ESCC. Our results also showed that gender and pathology stage were two independent prognostic factors for ESCC patients. Jung et al. [29] previously found that female cancer patients exhibited better survival for tumors of the head/neck, esophagus, small intestine, liver, nasal cavities, and lung compared to male patients, even after adjustment for age and stage. Our study also showed a better survival rate for patients with stage I and stage II ESCC compared with previous studies; this may due to scheduled examination and timely treatment after relapse in our center.

Our study has certain limitations. First, the size of the study group was relatively small. Second, a more precise and specific detection method should be applied to determine accurate serum sCLU concentrations. Third, 7 patients were lost to follow-up in our study; this considerably decreased our amount of data and may have affected the final results.

## 5. Conclusions

In conclusion, our study revealed that there was overexpression of sCLU in the serum of ESCC patients and that sCLU might be taken as a tumor marker in ESCC screening. Moreover, a lower clusterin concentration in ESCC patients correlates with poorer prognosis. Thus, clusterin may play a key role during tumorigenesis and tumor progression of ESCC and it could be applied in clinical work as a tumor marker and prognostic factor.

## Figures and Tables

**Table 1:** Patient characteristics.

Characteristics	Number (%)
Gender	
Male	79 (91)
Female	8 (9)
Age (yr)	
Median	60
Average	59
Tumor location	
Upper	5 (6)
Middle	58 (66)
Lower	25 (28)
Pathological stage	
I	13 (15)
II	35 (40)
III	39 (45)
Lymph node yield	
Median	21
Average	24

**Table 2:** Serum clusterin concentrations in ESCC patients and healthy individuals.

Variable	Number	Concentration (*μ*g/mL)	*P* value
ESCC patients	87	412.3 ± 159.4	<0.0001^*^
Healthy individuals	136	288.8 ± 75.1	
ESCC patients (excluding smoker)	30	419.4 ± 154.2	<0.0001^*^
Healthy individuals	136	288.8 ± 75.1	
ESCC patients (excluding drinker)	45	413.3 ± 141.9	<0.0001^*^
Healthy individuals	136	288.8 ± 75.1	
ESCC patients (excluding diabetes patients)	80	416.3 ± 164.1	<0.0001^*^
Healthy individuals	136	288.8 ± 75.1	
ESCC patients (excluding cardiovascular diseases patients)	73	406.88 ± 167.7	<0.0001^*^
Healthy individuals	136	288.8 ± 75.1	
ESCC patients (excluding all confounders)	17	400.5 ± 176.2	0.0028^*^
Healthy individuals	136	288.8 ± 75.1	

ESSC: esophageal squamous cell carcinoma.

^*^The *P* value describes the difference in serum clusterin concentration between the samples from the ESCC patients and those from the healthy controls. Significance was determined by independent-samples Mann-Whitney *U* test.

**Table:** (a) Univariate analysis

Factor	*P* value
Age	0.897
Gender (male versus female)	0.102
Tumor location	0.460
Upper versus lower	0.813
Middle versus lower	0.215
Pathological stage	0.001
Stage I versus stage III	0.037
Stage II versus stage III	0.001
Lymph node metastasis number	0.009
Lymph node yield Clusterin concentration (<500 versus >500 *μ*g/mL)	0.525 0.392

**Table:** (b) Multivariate analysis

Factor	*P* value	HR	95% CI
Lymph node metastasis number	0.517	0.919	0.847–1.807
Gender (male versus female)	0.040	8.466	1.106–64.800
Pathological stage	<0.0001		
Stage I versus III	0.006	0.113	0.024–0.538
Stage II versus III	<0.0001	0.145	0.050–0.418
Clusterin concentration (<500 versus >500 *μ*g/mL)	0.030	2.835	1.107–7.263

ESSC: esophageal squamous cell carcinoma; HR: hazard ratio; CI: confidence interval.

## Abbreviations

ESCC: Esophageal squamous cell carcinoma
sCLU: Secreted clusterin
nCLU: Nuclear clusterin
ELISA: Enzyme-linked immune sorbent assay
MMP: Matrix metalloproteinase
ROC: Receiver operating characteristic
AUROC: Area under the ROC curve.
